# Cannabinoid receptor 2 as a potential therapeutic target in rheumatoid arthritis

**DOI:** 10.1186/1471-2474-15-275

**Published:** 2014-08-12

**Authors:** Shin Fukuda, Hitoshi Kohsaka, Aiko Takayasu, Waka Yokoyama, Chie Miyabe, Yoshishige Miyabe, Masayoshi Harigai, Nobuyuki Miyasaka, Toshihiro Nanki

**Affiliations:** Department of Medicine and Rheumatology, Graduate School of Medical and Dental Sciences, Tokyo Medical and Dental University, 1-5-45, Yushima, 113-8519 Bunkyo-ku, Tokyo, Japan; Department of Dermatology, Tokyo Medical University, 6-1-1 Shinjuku, 160-8402 Shinjuku-ku, Tokyo Japan; Department of Pharmacovigilance, Graduate School of Medical and Dental Sciences, Tokyo Medical and Dental University, 1-5-45, Yushima, 113-8519 Bunkyo-ku, Tokyo, Japan; Department of Clinical Research Medicine, Teikyo University, 2-11-1 Kaga, 173-8605 Itabashi-ku, Tokyo, Japan

**Keywords:** Cannabinoid, Cannabinoid receptor 2 (CB_2_), Rheumatoid arthritis, JWH133, Fibroblast-like synoviocyte, Monocyte

## Abstract

**Background:**

Some of cannabinoids, which are chemical compounds contained in marijuana, are immunosuppressive. One of the receptors, CB receptor 1 (CB_1_), is expressed predominantly by the cells in the central nervous system, whereas CB receptor 2 (CB_2_) is expressed primarily by immune cells. Theoretically, selective CB_2_ agonists should be devoid of psychoactive effects. In this study, we investigated therapeutic effects of a selective CB_2_ agonist on arthritis.

**Methods:**

The expression of CB_2_ was analyzed with immunohistochemistry and Western blotting. Interleukin (IL)-6, matrix metalloproteinase-3 (MMP-3), and chemokine (C-C motif) ligand 2 (CCL2) were quantified with enzyme-linked immunosorbent assays (ELISA). Osteoclastogenesis was assessed with tartrate-resistant acid phosphatase staining and the resorption of coated-calcium phosphate. Effect of JWH133, a selective CB_2_ agonist, on murine collagen type II (CII)-induced arthritis (CIA) was evaluated with arthritis score, and histological and radiographic changes. IFN-γ and IL-17 production by CII-stimulated splenocytes and serum anti-CII Ab were analyzed by ELISA.

**Results:**

Immunohistochemistry showed that CB_2_ was expressed more in the synovial tissues from the rheumatoid joints than in those from the osteoarthritis joints. CB_2_ expression on RA FLS was confirmed with Western blot analysis. JWH133 inhibited IL-6, MMP-3, and CCL2 production from tumor necrosis factor-α-stimulated fibroblast-like synoviocytes (FLS) derived from the rheumatoid joints, and osteoclastogenesis of peripheral blood monocytes. Administration of JWH133 to CIA mice reduced the arthritis score, inflammatory cell infiltration, bone destruction, and anti-CII IgG1 production.

**Conclusion:**

The present study suggests that a selective CB_2_ agonist could be a new therapy for RA that inhibits production of inflammatory mediators from FLS, and osteoclastogenesis.

## Background

Rheumatoid arthritis (RA) is a systemic autoimmune disease of unknown etiology. It is associated with chronic inflammation, bone destruction in multiple joints, and various extra-articular manifestations. Unless treated properly, it is generally progressive with functional decline, significant morbidity, premature mortality, and socioeconomic costs [1]. Recently, biological agents, represented by anti-tumor necrosis factor (TNF) monoclonal antibodies (mAb), have been used widely to improve arthritis and to inhibit bone destruction. However, there remain patients who do not respond satisfactorily. While pain control is a significant issue for the patients, disease-modifying antirheumatic drugs (DMARDs) do not have immediate effects for pain relief. The patients have to depend on corticosteroids or non-steroidal anti-inflammatory drugs (NSAIDs).

Cannabinoids are pharmacologically active components of *Cannabis sativa*. The endogenous ligands for cannabinoid receptors represented by anandamide and 2-arachidonoylglycerol also occur as endocannabinoids. This system regulates various physiological processes such as appetite control, pain perception and immune responses. Cannabinoids transmit signals through cannabinoid (CB) receptors, CB receptor 1 (CB_1_) and CB receptor 2 (CB_2_) [2, 3]. CB_1_ is expressed predominantly by the cells in the central nervous system (CNS), whereas CB_2_ is expressed primarily by immune cells [2, 3] and by peripheral nerve terminals [4]. Recently, G-coupled receptor 55 (GPR55) was proved to be a receptor of many endocannabinoids [5, 6]. It is expressed ubiquitously in many organ systems, including CNS [7]. Furthermore, endocannabinoids act as agonists of transient receptor potential vanilloid type-1 and type-4 (TRPV-1, -4) [8, 9], and nuclear peroxisome proliferator-activated receptors (PPARs) [10]. Because of the potential effects on a wide variety of the receptors in the various organs, cannabinoids have not been accepted as therapeutic agents. Especially, the major concerns are their psychoactive effects such as catalepsy and hypolocomotion. In this regards, selective CB_2_ agonists should be devoid of psychoactive activities.

Immune cells sensitive to cannabinoids are macrophages, natural killer cells, T cells, and B cells [11]. Some cannabinoids were applied to treat collagen-induced arthritis (CIA), which is a murine model of RA. HU320, a synthetic cannabinoid ameliorated established CIA [12]. Since HU320 has no affinity for CB_1_ or CB_2_, its therapeutic effect on CIA may derive from actions on other receptors. Blockade of fatty acid amide hydrolase (FAAH), which is the primary degradative enzyme of anandamide, and acts as an agonist for CB_1_, CB_2_ and other receptors, reduced the severity of CIA [13]. However, therapeutic effect of selective CB_2_ agonists on animal models of RA has not been investigated.

It was reported that local administration of JWH133, which is a selective CB_2_ agonist with 200-fold selectivity for CB_2_ over CB_1_ (Ki values are 3.4 and 677 nM respectively) [14], inhibited pain reaction of mice with carrageenan-injected paws [15]. NSAIDs and corticosteroids have several toxicities especially in long-term treatment or at high doses. In many cases, opioid analgesics would be better choice for avoiding the toxicities. Add-on therapy for RA patients with tramadol, which is a weak opioid analgesic and acetaminophen combination tablet, significantly improved joint pain without severe adverse effects [16]. Thus, selective CB_2_ agonists would be expected not only to relief pain, but also to suppress arthritis.

In the present study, we investigated the expression of CB_2_ in the RA synovial tissues, *in vitro* effects of JWH133 on RA fibroblast-like synoviocytes (FLS) and human monocytes, and its *in vivo* therapeutic effects on CIA.

## Methods

### Specimens

Synovial tissue samples were obtained from seven RA patients, who fulfilled the ACR classification criteria [17] and three osteoarthritis (OA) patients undergoing total knee joint replacement. The RA patients were 62 (38-75) years old [median (range)], with a disease duration of 9 (3-15) years and C-reactive protein level of 4.6 (0.3-81) mg/l. Informed consent was obtained from all the patients. All experimental protocols were approved by the Ethics Committee of Tokyo Medical and Dental University.

### Immunohistochemistry

Immunohistochemical analysis was conducted on formalin-fixed paraffin-embedded sections of synovial tissues. The sections were incubated overnight at 4°C with 2 μg/ml rabbit anti-CB_2_ polyclonal antibody (pAb) (abcam, Cambridge MA) or normal rabbit IgG as a control. Subsequently, the samples were incubated with 2 μg/ml biotinylated goat anti-rabbit IgG (Santa Cruz Biotechnology, Dallas, TX) for 30 min at room temperature, and then incubated for 30 min with streptavidin–horseradish peroxidase (Dako, Glostrup, Denmark). Diaminobenzidine (Dako) was used for visualization. The sections were counterstained with hematoxylin. The CB_2_ staining of three RA and three OA samples was semi-quantitatively evaluated by randomly selected three fields with scored as follows: 0 = none, 1 = focal, and 2 = diffuse. The maximum score was six for each sample.

For immunofluorescence double-staining with CD68, CD4, CD8, CD21 or vimentin, and CB_2_, the sections were incubated overnight at 4°C with 2 μg/ml rabbit anti-CB_2_ pAb or normal rabbit IgG together with 1 μg/ml mouse anti-CD68 mAb (KP1; Dako), 1 μg/ml mouse anti-CD4 mAb (RPA-T4; eBioscience, San Diego, CA), 1 μg/ml mouse anti-CD8 mAb (HIT8a; BD Bioscience, San Diego, CA), 1 μg/ml mouse anti-CD21 mAb (1 F8; Dako) or 1 μg/ml mouse anti-vimentin mAb (V9; Dako). Subsequently, the samples were incubated with 2 μg/ml Alexa Fluor 488-conjugated goat anti-mouse IgG1 (Invitrogen, Grand Island, NY) and Alexa Fluor 568-conjugated goat anti-rabbit IgG1 (Invitrogen) for 30 min at room temperature. A nuclear stain was performed with 4’, 6-diamidino-2-phenylindole.

### Protein detection in cultured FLS

FLS from the RA synovial tissues was cultured as was reported previously [18]. RA FLS was lysed with radioimmunoprecipitation assay buffer (Millipore, Billerica, MA, USA) for 30 min at 4°C. A total of 20 μg of protein were boiled in the presence of sodium dodecyl sulfate (SDS) sample buffer and separated on a 10% SDS-polyacrylamide gel (ATTO, Tokyo, Japan). Proteins were then electrotransferred onto a polyvinylidene fluoride microporous membrane (Millipore) in a semidry system. The membrane was blocked with Block Ace (Snow Brand Milk Products, Tokyo, Japan) for 1 h at room temperature, and then the immunoblots were incubated overnight with 1 μg/ml rabbit anti-CB_2_ pAb in Can Get Signal Immunoreaction Enhancer Solution (Toyobo, Osaka, Japan) at 4°C. After washing, the immunoblots were incubated with 2 μg/ml biotinylated goat anti-rabbit IgG for 30 min at room temperature, and then incubated for 30 min with streptavidin–horseradish peroxidase. ECL Prime detection reagent and the ImageQuant LAS 4000 Mini Biomolecular Imager (both from GE Healthcare) were used to detect the bands.

RA FLS (1 × 10^5^ cells/ml) was cultured in Dulbecco's Modified Eagle Medium (Sigma-Aldrich) + 10% fetal bovine serum (FBS) (Sigma-Aldrich) and stimulated with 5 ng/ml recombinant TNF-α (R&D Systems, Minneapolis, MN) for 24 h in the presence or absence of JWH133 (Tocris bioscience, Ellisville, MO) [14]. The concentrations of Interleukin (IL)-6, metalloproteinase-3 (MMP-3) and chemokine (C-C motif) ligand 2 (CCL2) in the culture supernatants were measured using enzyme-linked immunosorbent assay (ELISA) kits (DuoSet; R&D Systems).

### Analysis of osteoclastogenesis

Peripheral blood mononuclear cells from healthy donors were collected using Ficoll-Conray (Imuuno-Biological Laboratories, Gunma, Japan) gradient centrifugation. Positive selection of CD14^+^ monocytes was performed using CD14 MicroBeads (Miltenyi Biotec, Auburn, CA). The purified peripheral blood CD14^+^ monocytes (1 × 10^6^ cells/well) were incubated in 96-well plates in α-Minimum Essential Medium (Sigma-Aldrich) with 10% FBS, and incubated with 25 ng/ml macrophage colony-stimulated factor (M-CSF) (R&D systems) + 40 ng/ml receptor activator of nuclear factor kappa-B ligand (RANKL) (Peprotech, Rocky Hill, NJ). These cells incubated in the presence or absence of JWH133. The medium was replaced with fresh medium 3 days later, and after incubation for 7 days the cells were stained for tartrate-resistant acid phosphatase (TRAP) expression using a commercial kit (Hokudo, Sapporo, Japan). The number of TRAP-positive multinucleated cells (MNC: more than 3 nuclear) in a randomly selected field examined at ×40 magnification was counted under light microscopy. The CD14^+^ monocytes were seeded onto plates coated with calcium phosphate thin films (Osteo Assay Plate, Corning, NY, USA) and were incubated with 25 ng/ml M-CSF + 40 ng/ml RANKL for 7 days in the presence or absence of JWH133. The cells were then lysed in bleach solution (6% NaOCl, 5.2% NaCl). The resorption lacunae were examined under light microscopy. The viability of the cells treated with JWH133 (up to 50 μM) was more than 95% relative to the vehicle-treated cells.

### Induction of collagen-induced arthritis (CIA)

Male 8-week-old DBA/1 J mice were purchased from Oriental Yeast (Tokyo, Japan) and were kept in the temperature of 23.5 ± 2 degrees Celsius with 40-50% humidity. Bovine collagen type II (CII; Collagen Research Center, Tokyo, Japan) was dissolved in 0.05 M acetic acid at 4 mg/ml and emulsified in equal volume of complete Freund’s adjuvant (CFA; Difco Laboratories, Detroit, MI). Mice were immunized with 100 μl of the emulsion injected intracutaneously at the base of the tail (day 1). After 21 days (day 22), the same amount of bovine CII emulsified in CFA was injected intracutaneously at the base of the tail as a booster immunization [19].

### Treatment of collagen-induced arthritis (CIA) mice with JWH133

Twelve mice with CIA per group were twice daily injected intraperitoneally with JWH133, 1 mg/kg/day or 4 mg/kg/day in total volume of 200 μl/day of 20% dimethyl sulphoxide or vehicle alone from day 15 to day 35. To determine the therapeutic effects of JWH133, we also treated mice with 4 mg/kg JWH133 from day 28, after the development of arthritis, to day 35 and observed the mice for signs of arthritis. Disease severity for each limb was recorded as follows: 0 = normal, 1 = erythema and swelling of one digit, 2 = erythema and swelling of two digits or erythema and swelling of ankle joint, 3 = erythema and swelling of more than three digits or swelling of two digits and ankle joint, and 4 = erythema and severe swelling of the ankle, foot, and digits with deformity. The clinical arthritis score was defined as the sum of the scores for all 4 paws of each mouse. Thickness of each paw was measured using a pair of digital slide calipers. On day 36, the ankle joints were harvested and examined radiographically and histologically. The bilateral second-to-fourth metatarsophalangeal (MTP) joints were assessed radiographically as follows: 0 = not obvious, 1 = marginal osteoporosis, and 2 = erosion. This system yields a possible score between 0 and 4 per animal. The hind paw of each mouse was dissected and examined histologically after hematoxylin and eosin staining. The severity of arthritis was evaluated according to synovial inflammation, as follows: 0 = no inflammation, 1 = focal inflammatory infiltration, and 2 = severe and diffuse inflammatory infiltration. The experimental protocol was approved by the Institutional Animal Care and Use Committee of Tokyo Medical and Dental University.

The harvested splenocytes (1 × 10^6^ cells) on day 36 were cultured in 48-well plates in Roswell Park Memorial Institute 1640 medium (Sigma-Aldrich) with 10% FBS supplemented with 50 μg/ml denatured (100°C, 10 min) CΙΙ. After 72 h, the concentrations of interferon (IFN)-γ and IL-17 in the culture supernatant were measured using ELISA kits (DuoSet; R&D Systems).

Serum samples were obtained on day 36 for measurement of IgG1 anti-CII antibody by ELISA (normal, n = 4; vehicle, n = 12; 4 mg/kg JWH133, n = 12) as described previously [19].

### Statistical analysis

All data are expressed as the mean ± standard error of the mean (SEM). Immunohistological score was analyzed by student’s t-test. Concentration of inflammatory mediators and osteoclastogenesis were analyzed by Kruskal-Wallis test and Dunnett’s test. Overtime analysis of arthritis score and paw thickness was performed by 2 way-ANOVA, and point-by-point analysis was followed by student’s t-test. Histological and radiographic score, and concentration of IgG1 were analyzed by Kruskal-Wallis test and student’s t-test.

## Results

### CB_2_ expression in the RA synovial tissues and cells

**Figure 1:**
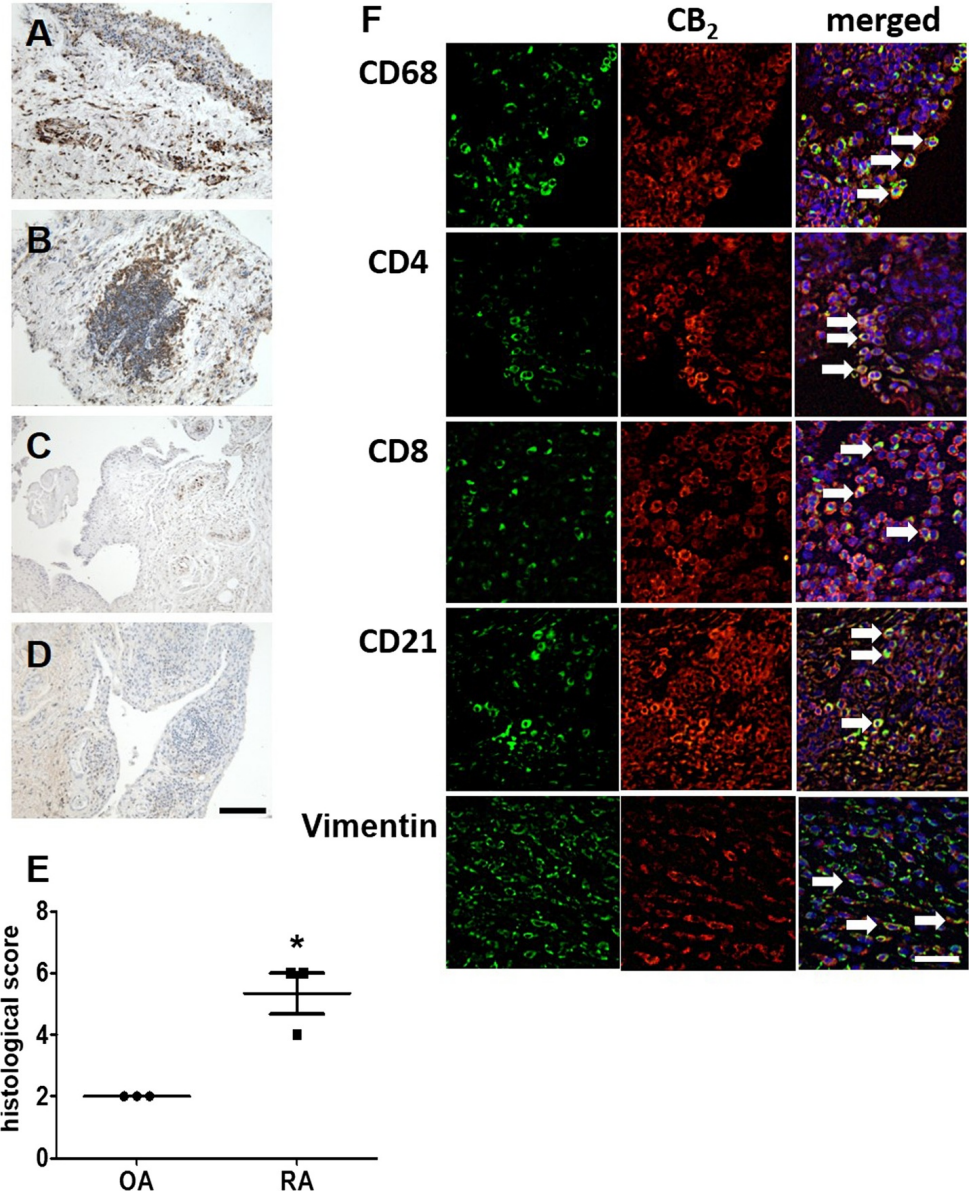
**CB**_**2**_ **expression in the RA synovial tissues.** Synovial tissue samples from patients with RA **(A, B, D)** or with OA **(C)** were stained with anti-CB_2_ pAb **(A B, and C)**, or normal rabbit IgG **(D)**. All sections were counterstained with hematoxylin. Bar, 400 μm. The representative figures of three RA and three OA synovial tissues are depicted. The severity of the CB_2_ staining of samples was shown **(E)**. Double immunofluorescence staining of the RA synovial tissues with CB_2_ and CD68, CD4, CD8, CD21 or vimentin, and DAPI are shown **(F)**. Arrows indicate double-positive cells in the merged image. Bar, 40 μm. *p < 0.05.

The RA and OA synovial tissues were examined for CB_2_ expression with immunohistochemical staining. We found positive staining in the lining, and sub-lining layer (Figure 1A), and follicle-like aggregates (Figure 1B). In contrast, minimal staining was observed in the OA synovial tissues (Figure 1C). No signal was observed on specimens stained with an isotype-matched IgG control of irrelevant specificity (Figure 1D). The CB_2_ staining of 3 RA and 3 OA samples was evaluated with immunohistological score. The scores of CB_2_ staining of RA samples was significantly higher than that of OA samples (Figure 1E).

In consistence with the fact that macrophages and lymphocytes express CB_2_ [20, 21], immunofluorescence double staining of the RA synovial tissues revealed CB_2_ expression on the synovial CD68^+^ macrophages, CD4^+^ T cells, CD8^+^ T cells, CD21^+^ B cells and vimentin^+^ fibroblast-like appearance cells (Figure 1F).

### Inhibition of inflammatory mediator production from RA FLS with a selective CB_2_ agonist

**Figure 2:**
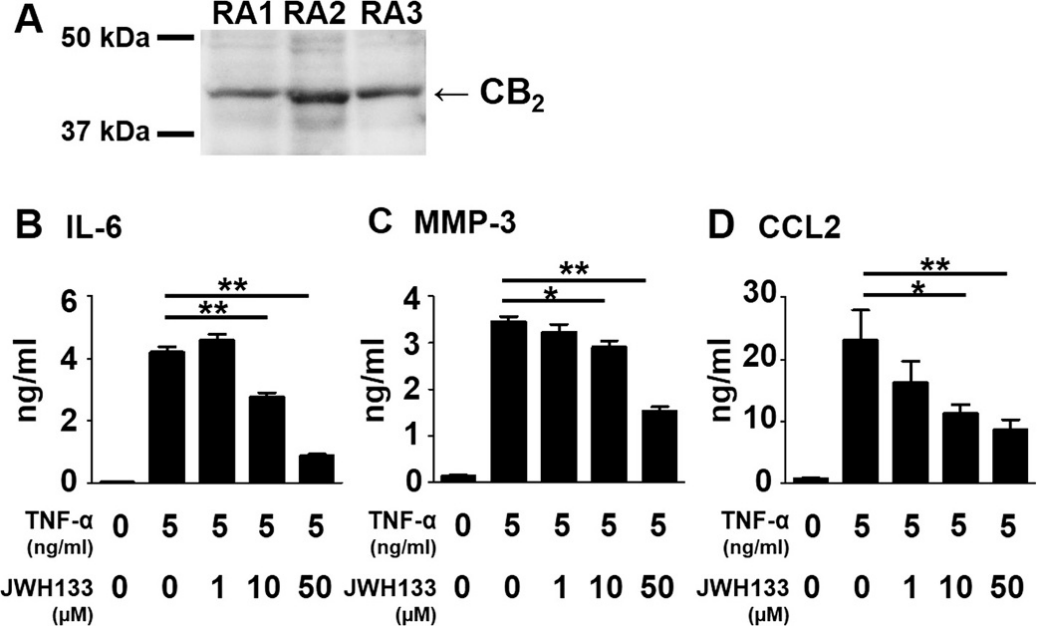
**Inhibition of IL-6, MMP-3, and CCL2 production from FLS by the selective CB**_**2**_ **agonist JWH133.** CB_2_ expression on *in vitro* cultured FLS from three RA patients was detected with Western blotting analysis **(A)**. Bars indicate 50 and 37 kDa. Predicted molecular weight of CB_2_ is 45 kDa. RA FLS were treated with various concentrations of JWH133, from 30 minutes before the stimulation with TNF-α for 24 h. Concentrations of IL-6 **(B)**, MMP-3 **(C)**, and CCL2 **(D)** in the cultured supernatants were measured with ELISA. Data is presented as means ± SEM of one of three independent experiments analyzed in quadruplicate. *p < 0.05, **p < 0.01.

FLS produce various inflammatory mediators that play important roles in development of RA. CB_2_ expression on *in vitro* cultured RA FLS was confirmed with Western blot analysis (Figure 2A), which agreed with previous observation [22]. We evaluated effects of JWH133, a selective CB_2_ agonist, on the production of IL-6, MMP-3, and CCL2 from FLS stimulated with TNF-α. TNF-α treatment enhanced production of these mediators from RA FLS, which was suppressed by JWH133 dose-dependently (Figure 2B-D). Treatment with JWH133 did not inhibit the proliferation of FLS evaluated with the cell counting kit (Dojindo, Kumamoto, Japan) using WST-8, 2-(2-methoxy-4-nitrophenyl)-3-(4-nitrophenyl)-5-(2,4-disulfophenyl)-2H-tetrazolium (data not shown).

### Inhibition of osteoclastogenesis of peripheral blood monocytes with a selective CB2 agonist

**Figure 3:**
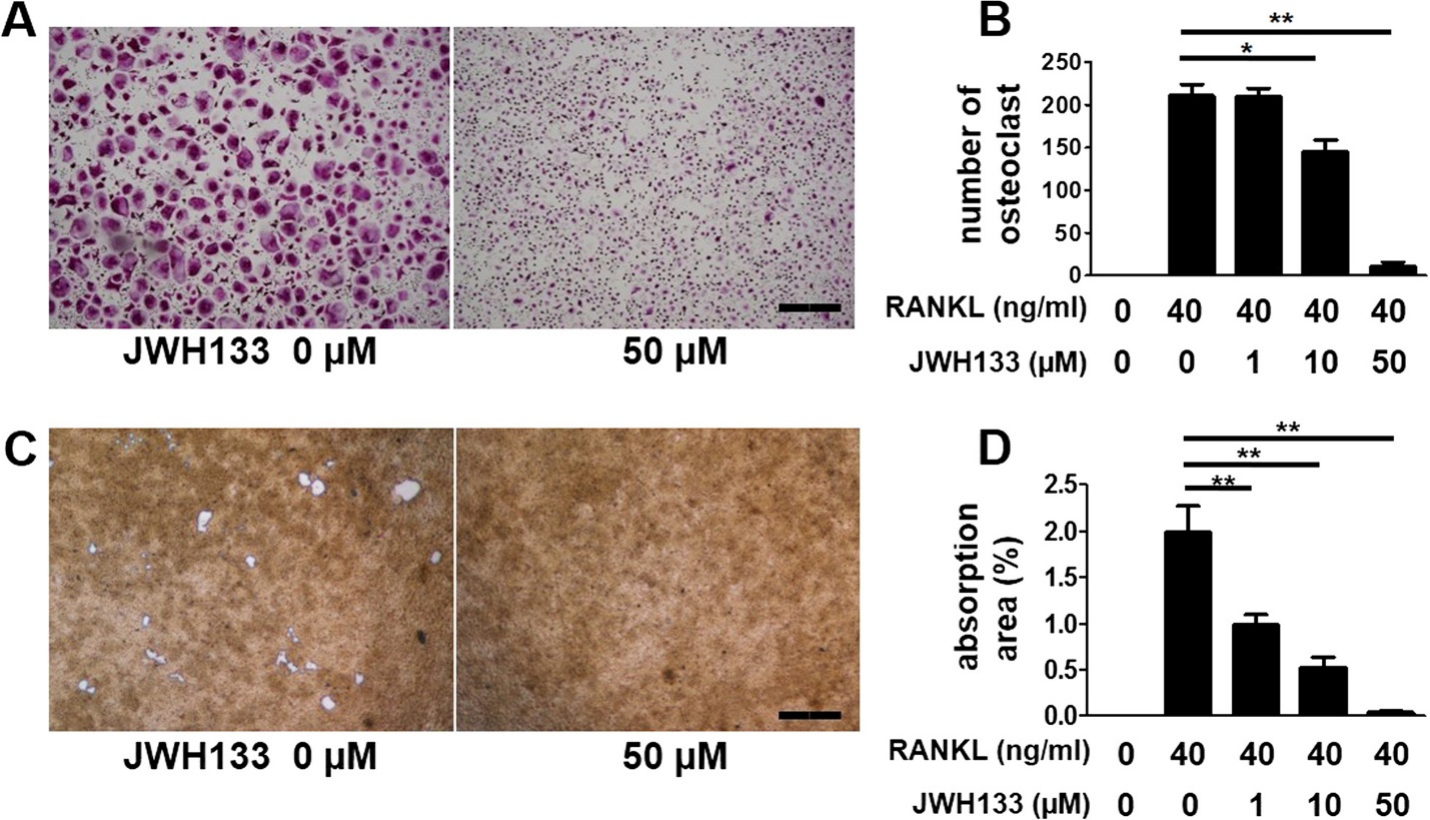
**Effects of the selective CB**_**2**_ **agonist JWH133 on peripheral blood monocytes.** Peripheral blood monocytes were treated with various concentrations of JWH133, from 30 minutes before the stimulation with M-CSF and RANKL. Cells were stained with TRAP **(A)**. TRAP-positive multinucleated cells in a randomly selected field examined at ×40 magnification were counted **(B)**. Representative data (mean ± SEM) from one of three independent experiments analyzed in triplicate are shown. The osteoclasts were incubated on calcium phosphate-coated plates **(C)**. The area of resorption lacunae was examined under light microscopy **(D)**. Representative data (mean ± SEM) from one of three independent experiments analyzed in triplicate are shown. Bar, 400 μm. *p < 0.05, **p < 0.01.

In the following experiments, we used human peripheral blood CD14^+^ monocytes, which have been shown to express CB_2_ [23], since it is hard to prepare a large number of synovial macrophages. Incubation of peripheral blood CD14^+^ monocytes with M-CSF and RANKL promoted development of TRAP-positive multinucleated cells. Co-incubation with JWH133 suppressed osteoclast formation dose-dependently (Figure 3A and B). M-CSF and RANKL treatment induced resorption of coated calcium. This was inhibited by the co-incubation with JWH133 dose-dependently (Figure 3C and D). These data showed that JWH133 inhibited the formation and function of human osteoclasts.

### Treatment of murine CIA with JWH133

**Figure 4:**
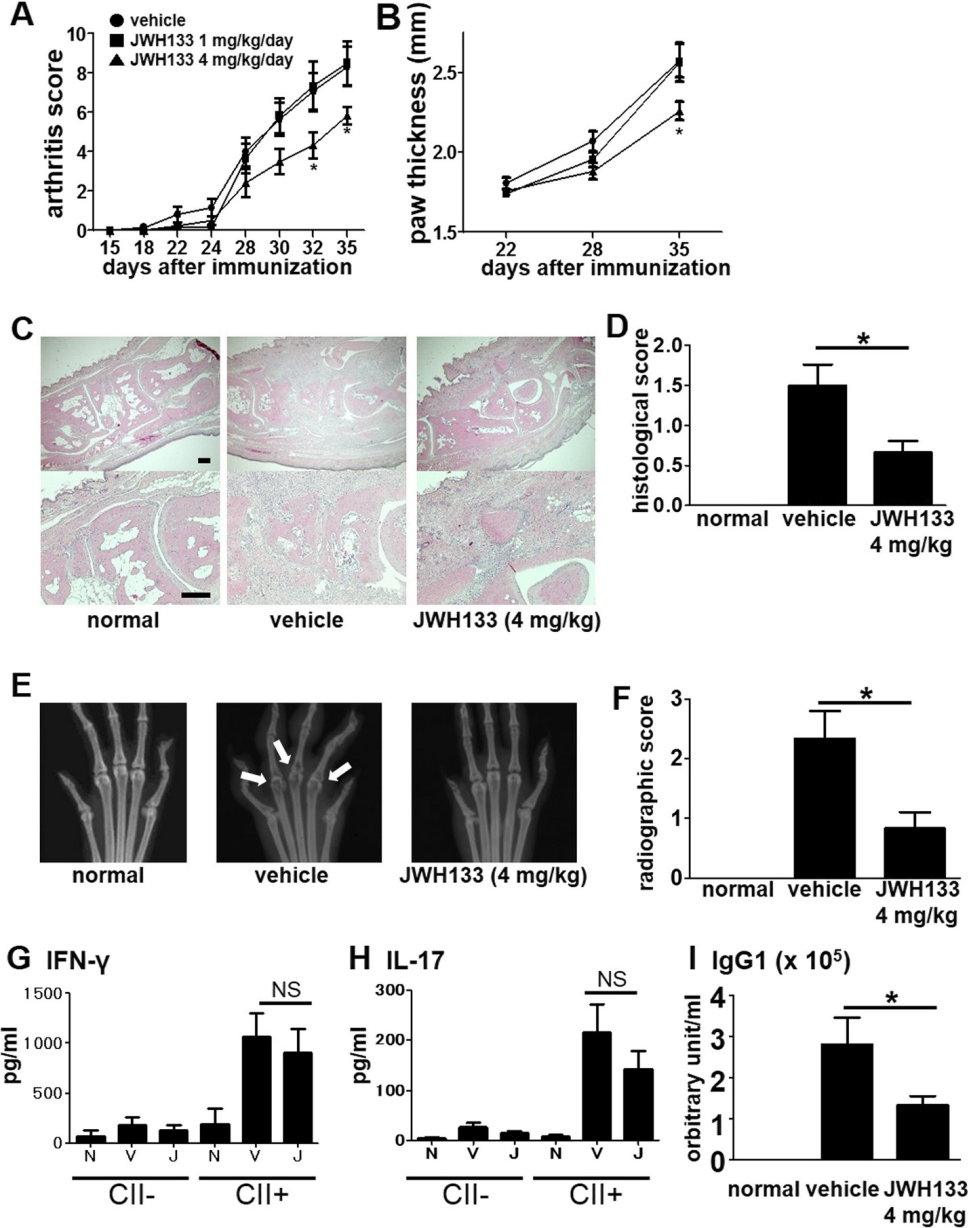
**Effects of the selective CB**_**2**_ **agonist, JWH133 on CIA mice.** JWH133 (1 mg/kg/day or 4 mg/kg/day) or vehicle (n = 12 each) was injected intraperitoneally twice daily from day 15 to day 35. The arthritis severity was recorded as the arthritis score **(A)** and paw thickness **(B)**. Overtime analysis of arthritis score and paw thickness by 2-way ANOVA revealed significant difference between vehicle group and 4 mg/kg/day grope (p < 0.01). Representative hematoxylin and eosin staining of ankle joints from normal mice and from mice with CIA after the treatment with vehicle or JWH133 (4 mg/kg/day) are shown **(C)**. Bar, 200 μm. Inflammatory cell infiltration in the ankle joints was evaluated with the histological score **(D)**. Representative radiographs of the ankle joints of normal mice and CIA mice treated with vehicle or JWH133 (4 mg/kg/day) **(E)**. Arrows indicate bone erosion. Bone erosion in the bilateral MTP joints was evaluated with the bone destruction score **(F)**. Splenocytes from normal mice and CIA mice treated with vehicle or JWH133 (4 mg/kg/day) were cultured with CII for 72 h. Concentrations of IFN-γ **(G)** and IL-17 **(H)** in the cultured supernatant were measured by ELISA. N: normal, V: vehicle, J: JWH133, NS: not significant. Serum samples were obtained at day 36 from normal mice and CIA mice treated with vehicle or JWH133 (4 mg/kg/day), and anti-CII IgG1 antibody level was measured by ELISA **(I)**. Values are the mean ± SEM. *p < 0.05 versus vehicle.

The inhibitory effects of JWH133 on RA FLS and human osteoclasts prompted us to examine the compound for the inhibitory effects on murine CIA, an animal model of RA. Intraperitoneal administration of JWH133 (1 mg/kg/day or 4 mg/kg/day) or vehicle twice daily was initiated two weeks after the first immunization and continued for 21 days. Treatment with JWH133 (4 mg/kg/day) ameliorated clinical severity of the arthritis (Figure 4A and B). However, the incidence of arthritis was 100% in all groups. On day 36, the ankle joints were harvested and examined histologically and radiographically. Cell infiltration in the synovial tissues and radiographical bone destruction were observed in vehicle-treated CIA mice, and reduced significantly in the JWH133-treated mice (Figure 4C,D,E and F).

Splenocytes from the mice at day 36 were stimulated with CII and the production of IFN-γ and IL-17 was measured. IFN-γ and IL-17 production was upregulated by the CII stimulation. The treatment with JWH133 did not alter the cytokine production (Figure 4G and H). To determine the effect of JWH133 on anti-CII antibody production, we measured serum anti-CII IgG1 antibody titer by ELISA on day 36. While anti-CII IgG1 antibody was not detected in normal mice, it was detected in CIA mice. The treatment with JWH133 significantly lowered anti-CII IgG1 antibody level (Figure 4I). Although IgG2a and IgG2b anti-CII antibodies were also detected in CIA mice, treatment with JWH133 did not significantly alter the levels of antibodies (data not shown).

**Figure 5:**
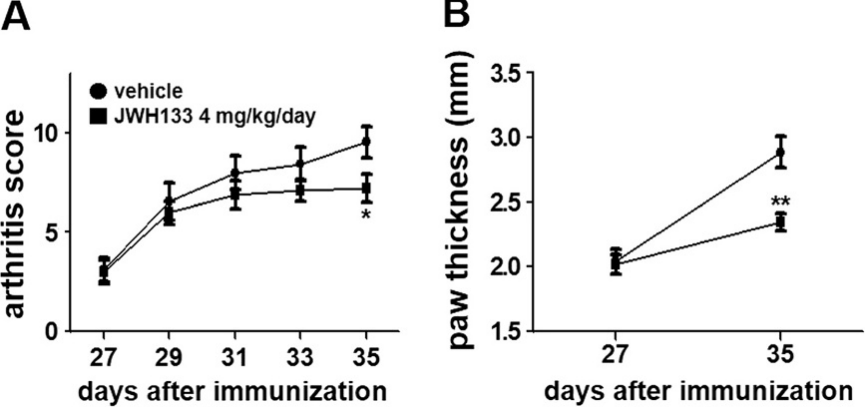
**Therapeutic effect of JWH133 on CIA.** CIA mice were treated with intraperitoneal injection of JWH133 (4 mg/kg/day) or vehicle (n=12 each) twice daily from day 28 to day 35, after the onset of the disease. The arthritis severity was recorded as the arthritis score **(A)** and paw thickness **(B)**. Overtime analysis of arthritis score and paw thickness by 2-way ANOVA revealed significant difference between vehicle group and 4 mg/kg/day grope (p < 0.01). Values are the mean ± SEM. *p < 0.05, **P < 0.01 versus vehicle.

To examine the therapeutic effects of JWH133 after the onset of the arthritis, we treated the CIA mice with 4 mg/kg JWH133 from day 28 to day 35, and observed inhibition of the arthritis (Figure 5A and B).

## Discussion

The present studies demonstrated that the selective CB_2_ agonist, JWH133 inhibited production of IL-6, MMP-3 and CCL2 from FLS and M-CSF and RANKL-induced osteoclastogenesis of monocytes/macrophages. Administration of JWH133 ameliorated arthritis severity and bone destruction, and decreased anti-CII IgG1 antibody in a murine model of RA. This effectiveness could be attributable to the suppression of inflammatory mediator secretion from FLS and osteoclastogenesis and autoantibody production. Thus, selective CB_2_ agonists could be a therapeutic agent for RA.

While medical use of cannabinoids such as sedation and analgesia was recorded in 19^th^ century [24], narcotic addiction was always concerned. Recent identification of cannabinoid receptors revealed two distinctive receptors, CB_1_ and CB_2_, and putative third receptor, GPR55. Some cannabinoids also act as agonists of TRPV-1, -4 and PPARs. It is expected that selective CB_2_ agonists act as therapeutic agents that modulate immune functions without any psychoactive effects. JWH133 was applied to our study since it has high selectivity for CB_2_ against CB_1_ and has no affinity for GPR55, TRPV-1, -4 or PPARs.

In RA patients, FLS expresses hyperplastic, inflammatory, cartilage- and bone-destructive phenotypes. Cytokines, chemokines and MMPs are secreted by FLS. It was reported that MMP-3 production from FLS stimulated with TNF-α or IL-1β was suppressed by ajulemic acid, a synthetic cannabinoid [22]. However, the receptors of this compound have not been identified. In this study, we showed that the selective CB_2_ agonist, JWH133 significantly reduced the production of IL-6, MMP-3, and CCL2 from FLS stimulated with TNF-α.

IL-6 has a wide range of functions on immune cells, and plays an important role in RA [25]. Blockade of IL-6 signaling by anti-IL-6 or -IL-6 receptor mAbs is effective treatment [26, 27]. Among various chemokines, only CCL2 gene expression is reportedly higher in RA FLS than in OA FLS [28]. Monocyte migration induced by RA stromal cell line supernatants was blocked with anti-CCL2 mAbs [29]. MMP-3 was highly expressed in the RA pannus tissues [30]. In the cytoplasm, CB_2_ stimulation leads to inhibition of adenylyl cyclase and subsequent decrease of the intracellular cAMP level. It results in decreased activity of protein kinase A and transcription factors such as NF-κB and NFAT [31, 32]. Thus, the treatment of JWH133 could suppress other inflammatory mediators than IL-6, CCL2 and MMP-3, which are involved in pathology of the arthritis.

JWH133 may affect other types of cells involved in the arthritis. RANTES/CCL5-induced chemotaxis of macrophages was inhibited with delta-9-tetrahydrocannabinol, the major component in *marijuana*, acting through CB_2_ [33]. Anandamide, one of endocannabinoids, suppressed IL-17 production from Th17 cells primarily via CB_2_ [34]. JWH015, another selective CB_2_ agonist, inhibited CXCL12-induced chemotaxis of T cells [35]. We found that CB_2_ was expressed broadly in the RA synovial cells including macrophages, T cells, and B cells. Suppression of these cells could contribute to suppression of the arthritis.

It was reported that CB_2_-deficient mice develop osteoporosis with age [36]. HU308, another selective CB_2_ agonist, inhibited osteoclast formation of RANKL-stimulated RAW264.7 cells as well as bone marrow cells from normal mice but not from CB_2_-deficient mice [36]. In agreement with this observation, we demonstrated that JWH133 inhibited osteoclastogenesis of human peripheral blood monocytes and bone destruction of CIA mice. Since bone destruction is often a serious issue in RA patients as well as chronic pain and can result in functional decline, inhibitory effect of selective CB_2_ agonists for osteoclastogenesis could be another feature in RA treatment.

In this study, we observed amelioration of CIA with JWH133. Since T helper cell differentiation influences the development of CIA [37, 38], we measured the production of IFN-γ and IL-17 by CII-stimulated splenocytes from the CIA mice. No significant difference among the groups was revealed in our study. It is suggested that the treatment with JWH133 did not affect Th1 and Th17 differentiation, which may not be attributable to the amelioration of CIA. On the other hand, the treatment with JWH133 decreased the level of serum anti-CII IgG1 antibody. In the previous study, CB_2_ mRNA was detected in peripheral B cells [20]. We determined CB_2_ expression on B cells in RA synovial tissue. Although the effect of CB_2_ for immunoglobulin production has not been reported, the administration of JWH133 directly or indirectly may affect B cells to suppress anti-CII IgG1 antibody production of CIA mice and contribute to the amelioration of the arthritis.

This is the first report of therapeutic effect of a selective CB_2_ agonist on CIA. Although the effect was mild, optimization of dosage and/or treatment protocol might enhance the effect. Perhaps, more potent selective CB_2_ agonists might solve this problem.

## Conclusions

We demonstrated that JWH133, the selective CB_2_ agonist, provides clinical effectiveness against CIA mice probably through the immunosuppressive effects for FLS and monocytes and inhibition of anti-CII Ab production. Addition to the analgesic effect as previously reported, selective CB_2_ agonists could be a new therapy for RA.

## Abbreviations

CB: Cannabinoid
CB_1_: Cannabinoid receptor 1
CB_2_: Cannabinoid receptor 2
CCL2: CC chemokine ligand 2
CFA: Complete Freund’s adjuvant
CIA: Collagen-induced arthritis
CII: Collagen type II
FLS: Fibroblast-like synoviocytes
M-CSF: Macrophage colony-stimulated factor
MMP: Matrix metalloproteinase
MNC: Multinucleated cells
OA: Osteoarthritis
RA: Rheumatoid arthritis
RANKL: Receptor activator of nuclear factor kappa-B ligand
TRAP: Tartrate-resistant acid phosphatase.
